# Supplementary material for: Content-rich biological network constructed by mining PubMed abstracts
Source: BMC Bioinformatics. 2004 Oct 8;5:147. doi: 10.1186/1471-2105-5-147 (PMC528731; doi:10.1186/1471-2105-5-147)
Supplement: Additional File 5 — The original Chilibot query results of the term "long-term potentiation (LTP)" and 22 other terms, limiting the latest references analyzed to the years 1990, 1995, 2000, and 2004. [file 1471-2105-5-147-S5.bz2 › chilibotAdditionalFile5/ltp2000/html/PI-3K_ACTIN.html]

 


 **PI-3K** and **ACTIN** 
  
Found 135 abstracts in PubMed,  **30 abstracts were retrieved and analyzed**.  


---

 Search Google  |
 PDF files only 
|  EDU domain only 

---

**Interactive relationship** (e.g. stimulation, inhibition, etc)

- beta 1 integrin and PI 3 kinase  [ **PI-3K** ]  regulate RhoA dependent activation of skeletal alpha  **actin**  promoter in myoblasts.  Ref: 10843867 Am J Physiol Heart Circ Physiol, 2000
- In summary, the p110alpha isoform of PI 3 kinase  [ **PI-3K** ]  is specifically required for EGF stimulated  **actin**  nucleation during lamellipod extension in breast cancer cells.  Ref: 10660520 J Biol Chem, 2000
- Thus, CrkII enhanced PI 3 kinase  [ **PI-3K** ]  activity is used to enhance  **actin**  filament reorganization in response to both hGH and hIGF 1, to enhance stress activated protein kinase activity in response to hGH, and to diminish STAT5 mediated transcription in response to hGH.  Ref: 10748058 J Biol Chem, 2000
- In addition, focal adhesion kinase FAK and phosphatidylinositol PI 3 kinase  [ **PI-3K** ]  were required for full activation of the alpha  **actin**  promoter by RhoA.  Ref: 10843867 Am J Physiol Heart Circ Physiol, 2000
- However, increased complex formation between 14 3 3 zeta and GP Ib IX and between PI 3 kinase  [ **PI-3K** ]  and GP Ib IX was detected in  **actin**  cytoskeletal fractions derived from thrombin or vWF stimulated platelets.  Ref: 10887121 Blood, 2000
- Our findings indicate that an intact  **actin**  network is a crucial requirement for PI 3 kinase  [ **PI-3K** ]  mediated production of 3 phosphoinositides and, therefore, for the activation of PKB.  Ref: 11104665 Biochem J, 2000
- PI 3 kinase  [ **PI-3K** ]  and erk 1 and 2 signaling pathways are activated and necessary for collagen binding and integrin mediated  **actin**  reorganization in embryonic avian corneal epithelium.  Ref: 11006227 Invest Ophthalmol Vis Sci, 2000
- Thus, the Src SH3 domain, which binds PI 3 kinase  [ **PI-3K** ]  and which is necessary for activation of Akt downstream, is required for the  **actin**  dependent targeting of v Src to focal adhesions.  Ref: 10938128 Mol Cell Biol, 2000
- **Actin**  depolymerization did not affect the hormonal activation of phosphoinositide 3 kinase PI 3 kinase  [ **PI-3K** ] , and since cytochalasin D treatment also led to reduced platelet derived growth factor PDGF induced phosphorylation of PKB in U87MG cells, a PTEN phosphatase and tensin homologue deleted on chromosome 10 null cell line, lipid phosphatase activity was unlikely to account for any reduction in cellular 3 phosphoinositides.  Ref: 11104665 Biochem J, 2000
- Moreover, Gbetagamma subunits and membrane targeted forms of phosphatidylinositol PI 3 kinase  [ **PI-3K** ]  alpha were sufficient to induce similar  **actin**  reorganization, and coexpression of various mutants of PI 3 kinase  [ **PI-3K** ]  with the N formyl peptide receptor identified a link to class Ia PI 3 kinase  [ **PI-3K** ]  mediated  **actin**  reorganization.  Ref: 10843992 J Biol Chem, 2000
- Erk and PI 3 kinase  [ **PI-3K** ]  are necessary for collagen binding and  **actin**  reorganization in corneal epithelia.  Ref: 11006227 Invest Ophthalmol Vis Sci, 2000
- Proposed downstream events in PI 3 kinase  [ **PI-3K** ]  regulated Arf cascades include modulation of vesicular trafficking and the  **actin**  cytoskeleton.  Ref: 11050434 Trends Biochem Sci, 2000
- This process requires both PI3 kinase  [ **PI-3K** ]  and MAP kinase kinase activity and depends on coordinated changes in the  **actin**  and microtubule based cytoskeleton.  Ref: 10702796 Oncogene, 2000
- During phagocytosis coronin is recruited together with PI 3 kinase  [ **PI-3K** ]  to membranes of nascent and early phagosomes co localizing with the  **actin**  cytoskeleton, confirming that coronin contributes to phagocytosis.  Ref: 11094157 FEBS Lett, 2000
- Expression of a dominant negative mutant of FAK, application of wortmannin to cultured myoblasts, or expression of a dominant negative mutant of PI 3 kinase  [ **PI-3K** ]  inhibited alpha  **actin**  promoter activity induced by RhoA.  Ref: 10843867 Am J Physiol Heart Circ Physiol, 2000
- Fluorescence staining of the cells with rhodamine conjugated phalloidin revealed rapid disassembly of  **actin**  filaments by cytochalasin B and leptin, the latter action being prevented by the phosphoinositide 3 PI 3 kinase  [ **PI-3K** ]  inhibitor LY 294002.  Ref: 10944173 J Physiol, 2000

**Parallel relationship** (e.g. studied together, co-existance, homology, etc.)

- FimH mediated invasion required localized host  **actin**  reorganization, phosphoinositide 3 kinase PI 3 kinase  [ **PI-3K** ]  activation and host protein tyrosine phosphorylation, but not activation of Src family tyrosine kinases.  Ref: 10856226 EMBO J, 2000
- Wortmannin, a phosphatidylinositol 3 kinase PI3 kinase  [ **PI-3K** ]  inhibitor that works as a second messenger for pp60c src, and cytochalasin B, an inhibitor of  **actin**  polymerization, suppressed the secretion of both CAK and CAL without suppressing synthesis.  Ref: 10872813 Biochem Biophys Res Commun, 2000
- In the current investigation specific inhibitors of PI 3 kinase  [ **PI-3K** ]  and mitogen activated kinase kinase MEK 1 kinase were used to determine the role of these signaling molecules in  **actin**  reorganization and collagen binding to isolated sheets of corneal epithelial tissue.  Ref: 11006227 Invest Ophthalmol Vis Sci, 2000
- The extension of lamellae requires net  **actin**  assembly and an exposure of  **actin**  filament barbed ends downstream of PI 3 kinase  [ **PI-3K** ] .  Ref: 11090061 Blood, 2000
- Collectively our results suggest that IGF I regulates SC motility by reorganization of the  **actin**  cytoskeleton via the downstream activation of a PI 3 kinase  [ **PI-3K** ] , small GTPase, and FAK pathway.  Ref: 10829021 J Biol Chem, 2000
- Changes in the  **actin**  cytoskeleton, controlled by H ras61L mediated activation of the Rac Rho pathway, as well as PI 3 kinase  [ **PI-3K** ]  activation, can also occur in the absence of C terminal lipid modifications.  Ref: 10854057 Exp Cell Res, 2000
- We have proposed that H Ras sensitive activation of phosphoinositide 3 PI 3 kinase  [ **PI-3K** ]  and subsequent profilin mediated  **actin**  polymerization, can be involved in chemokine induced integrin dependent adhesion of T cells.  Ref: 11005242 Histol Histopathol, 2000
- Changes in the distribution of f  **actin**  after PI3 kinase  [ **PI-3K** ]  inhibition suggest that this disruption of adherens junctions may be mediated by alterations to the cytoskeleton.  Ref: 11100902 Differentiation, 2000
- The tissues were evaluated for collagen stimulated  **actin**  reorganization, erk 1 and 2 and PI 3 kinase  [ **PI-3K** ]  activity, total filamentous  **actin**  accumulation, and collagen binding.  Ref: 11006227 Invest Ophthalmol Vis Sci, 2000
- Upstream of the WASP family proteins, receptor tyrosine kinases, G protein coupled receptors, phosphoinositide 3 OH kinase PI 3 kinase  [ **PI-3K** ] , and the Rho family of GTPases receive and transduce the signals that lead to  **actin**  nucleation through WASP Arp2 3 action.  Ref: 11073096 J Leukoc Biol, 2000
